# Genetic variants in cellular transport do not affect mesalamine response in ulcerative colitis

**DOI:** 10.1371/journal.pone.0192806

**Published:** 2018-03-26

**Authors:** Christopher J. Moran, Hailiang Huang, Manuel Rivas, Jess L. Kaplan, Mark J. Daly, Harland S. Winter

**Affiliations:** 1 Division of Pediatric Gastroenterology, Hepatology, & Nutrition, MassGeneral Hospital *for* Children, Boston, Massachusetts, United States of America; 2 Department of Pediatrics, Harvard Medical School, Boston, Massachusetts, United States of America; 3 Analytic and Translational Genetics Unit, Massachusetts General Hospital, Boston, MA, United States of America; 4 Broad Institute of MIT and Harvard, Cambridge, Massachusetts, United States of America; 5 Department of Biomedical Data Science, Stanford University, Stanford, California, United States of America; German Cancer Research Center (DKFZ), GERMANY

## Abstract

**Background and aims:**

Mesalamine is commonly used to treat ulcerative colitis (UC). Although mesalamine acts topically, in vitro data suggest that intracellular transport is required for its beneficial effect. Genetic variants in mucosal transport proteins may affect this uptake, but the clinical relevance of these variants has not been studied. The aim of this study was to determine whether variants in genes involved in cellular transport affect the response to mesalamine in UC.

**Methods:**

Subjects with UC from a 6-week clinical trial using multiple doses of mesalamine were genotyped using a genome-wide array that included common exome variants. Analysis focused on cellular transport gene variants with a minor allele frequency >5%. Mesalamine response was defined as improvement in Week 6 Physician’s Global Assessment (PGA) and non-response as a lack of improvement in Week 6 PGA. Quality control thresholds included an individual genotyping rate of >90%, SNP genotyping rate of >98%, and exclusion for subjects with cryptic relatedness. All included variants met Hardy-Weinberg equilibrium (p>0.001).

**Results:**

457 adults with UC were included with 280 responders and 177 non-responders. There were no common variants in transporter genes that were associated with response to mesalamine. The genetic risk score of responders was similar to that of non-responders (p = 0.18). Genome-wide variants demonstrating a trend towards mesalamine response included *ST8SIA5* (p = 1x10^-5^).

**Conclusions:**

Common transporter gene variants did not affect response to mesalamine in adult UC. The response to mesalamine may be due to rare genetic events or environmental factors such as the intestinal microbiome.

## Introduction

Ulcerative colitis (UC) is an inflammatory bowel disease (IBD) that predominantly affects the large intestine.[1] Medical treatment of UC depends on anti-inflammatory medications–some with systemic administration while others rely on topical delivery in the gastrointestinal tract. Although the pathogenesis of UC is not fully understood, it is clear that there is a strong genetic predisposition with >133 single nucleotide polymorphisms (SNPs) associated with the risk of developing UC.[2, 3] Similarly, genetic variants have been linked to medically-refractory disease and response to anti-tumor necrosis factor agents although the magnitude of these data is less than that linked to disease risk.[4–6]

Mesalamine is commonly-used in UC and exerts its anti-inflammatory effect topically with the active moiety delivered to the site of inflammation. Mesalamine is effective, but response rates in clinical trials of mesalamine are only 51–70%.[7–9] The action of mesalamine is not completely understood with possible mechanisms that include both epithelial cell-independent actions such as scavenging of luminal reactive oxygen species (ROS) and cell-dependent mechanisms such as inhibition of prostaglandin and leukotriene synthesis as well as blockade of cytokine-induced NFκB activation.[10, 11] The transport of mesalamine into the intracellular space has been shown to be a saturable process suggesting a transporter-mediated effect.[12] Further, Konig *et al* demonstrated in human embryonic kidney cells that epithelial transport of mesalamine was dependent on SLCO1B1, SLCO1B3, and SLCO2B1 (all of which are also expressed in intestinal cells) and that genetic variants in these genes diminish this transport.[13] Further, the metabolism of mesalamine into inactive form occurs by n-acetylation by NAT1 which also occurs in the intracellular space.[14]

Taken together, these studies suggest that mesalamine likely utilizes natural carrier transport proteins and that variation in these genes may have an effect on intracellular transport. However, the clinical impact of variation in transport genes on the efficacy of mesalamine has not been studied. The hypothesis of this study is that variation in genes involved in intracellular transport explains the variable response to mesalamine in patients with UC.

## Materials and methods

Subjects in this study were enrolled in the ASCEND III trial (ClinicalTrials.gov Identifier: NCT00350415), a 6 week double-blinded, randomized controlled trial of mesalamine (Asacol, Procter & Gamble Pharmaceuticals, Inc) to assess non-inferiority of two doses of mesalamine (4.8 grams versus 2.4 grams per day) in adults with moderately active UC.[9] Study trial entry required a diagnosis of UC based on standard clinical, endoscopic, histologic, and radiologic criteria. Subjects had their disease classified by the Montreal classification system.[15] The primary endpoint was symptomatic improvement or remission based on the “Physician’s Global Assessment” (PGA) as determined by study investigators that was based on rectal bleeding, stool frequency, and sigmoidoscopic assessment. Rectal bleeding assessment was characterized as: a lack of rectal bleeding, streaks of blood in the stool in <50% of stools, obvious blood with stool most of the time, and blood passed without stool. Stooling frequency was characterized as stool frequency normal for subject, 1–2 stools more than normal for subject, 3–4 stools more than normal for subject, and 5 or more stools greater than normal for subject. Sigmoidoscopic evaluation was characterized as: normal vasculature without friability, erythema with diminished vascular markings, marked erythema with contact bleeding and no ulcerations, and ulcerations with spontaneous bleeding.

In the current study, subjects from ASCEND III were classified as mesalamine responders if their Week 6 PGA improved from baseline (utilizing the original trial’s main efficacy outcome) and mesalamine non-responders if their Week 6 PGA did not improve. A stricter definition was utilized in secondary analysis that defined mesalamine responders as having both Week 6 PGA improvement and an improvement in the Week 3 rectal bleeding score (by at least one category).

### Genotyping

Genotyping was performed on the Illumina Infinium PsychArray-24 which contains 265,000 tag SNPs on the Infinium HumanCore-24 BeadChip and 245,000 markers from the Infinium Exome-24 BeadChip at the Broad Institute (Cambridge, MA). Primary analysis focused on genetic variants in genes involved in solute and drug transport. The list of transporter genes was further focused to only genes showing expression within the colonic mucosa based on expression profiles in Tissue specific Gene Expression and Regulation database (TiGER).[16] Utilization of the TiGER database restricted the genes of analysis to 47 (see S1 Table). Genetic variants were analyzed for variants with minor allele frequency (MAF) >5% in healthy controls as well as SNPs associated with UC by genome-wide association studies (GWAS).[2]

Genotype extraction was performed by PlinkV1.07.[17] Quality control procedures ensured that all included SNPs had a genotyping success rate of >98%, individual genotyping call rate >90%, and that they met the Hardy-Weinberg equilibrium threshold of p>0.001. For each SNP, the MAF was determined for mesalamine responders and mesalamine non-responders, and the odds ratio was calculated comparing these two groups.

Genetic risk score was calculated using Plink V1.07.[17] The score was composed of component risk scores at 133 UC-risk loci described by Jostins *et al*.[2] The component score (at each locus) was based on the log odds ratio for that SNP multiplied by the number of risk alleles that a subject possessed.[18] The component scores were then summed to generate a genetic risk score for each subject. Data from risk loci were based on either direct genotyping or data imputed from linked SNPs with r^2^>0.8.

### Statistical analysis

Demographics data was analyzed by student’s t test for continuous variables and Chi square analysis for categorical variables. Chi square analysis was performed on each SNP that passed quality control standards. P values threshold was set at <0.0013 to correct for multiple testing (based on 38 common variants in transporter genes that met quality control standards). A priori power calculations demonstrated that this sample size would have 85% power to demonstrate a 20% absolute difference in minor allele frequency between mesalamine responders and non-responders. To control for possible confounding variables in the cohort, the Cochran-Mantel-Haenszel test was applied on the 2x2 tables. Secondary analysis included all SNPs that passed quality control including non-coding SNPs and rare SNPs (MAF<5%) with p value threshold of 1x10^-8^.

The genetic risk score distribution of mesalamine responders and non-responders were compared by student’s t test. Subjects were assigned into genetic risk score quartiles and the response rates of the highest and lowest quartile were analyzed by Chi square analysis.

### Ethical considerations

DNA samples were obtained from ASCEND III participants under additional informed consent at the time of trial participation. Local IRB approval had been obtained for DNA sample collection. All procedures performed in studies involving human participants were in accordance with the ethical standards of the institutional and/or national research committee and with the 1964 Helsinki declaration and its later amendments or comparable ethical standards. All subjects involved were consented for DNA sample collection at the time of the original clinical trial enrollment at their respective institution after local ethics board review of the clinical trial protocol. The Partners Healthcare institutional review board approved the subsequent use of these DNA samples in this study (judging this project to be consistent with the original consent).

## Results

The cohort of 457 adult subjects with UC included 280 mesalamine responders and 177 mesalamine non-responders. As summarized in Table 1, the average age of the mesalamine responders was slightly older similar to the age of non-responders (44.2 vs. 41.6 years, p = 0.04) and both groups were predominantly white (97.8% vs. 97.6%, p = 0.88). Maximum disease distribution was more extensive in mesalamine non-responders (p = 0.027). A higher percentage of mesalamine-non-responders had been previously exposed to corticosteroids (47.9% vs. 32.6%, p = 0.0017) and immunomodulators (7.7% vs. 1.8%, p = 0.0046). Relapse frequency and disease duration were similar between the mesalamine responders and non-responders. The remainder of subject demographics and disease characteristics are shown in Table 1.

**Table 1:** Description of cohort under study. Disease distribution was defined by Paris classification system. All comparisons were made by Chi square analysis or student’s t test.

	Mesalamine Responders (n = 280)	Mesalamine Non-Responders (n = 177)	P value
Gender (% Male)	52.3%	60.9%	0.09
Average Age (Years)	44.2	41.6	0.04
Ethnicity:			
White:	97.8%	97.6%	0.88
Mesalamine Dose			
2.4g daily:	45.4%	53.7%	0.10
4.8g daily:	54.6%	46.3%	
Montreal Disease Distribution:			
E1:	55.1%	41.3%	
E2:	31.5%	40.1%	0.025
E3:	13.3%	18.5%	
Disease Duration:			
<1 Year:	27.2%	27.2%	
1–5 Years:	35.1%	35.5%	1.0
5–10 Years:	20.4%	20.1%	
>10 Years:	17.2%	17.2%	
Relapse Frequency:			
Newly Diagnosed:	17.5%	13.3%	
Once Monthly:	2.1%	5.1%	0.06
Once every 6 months:	21.4%	25.6%	
Once every 6–12 months	26.0%	21.8%	
Less than once every year:	12.5%	14.6%	
Past Medications:			
Corticosteroids:	32.6%	47.9%	0.0017
Immunomodulators:	1.8%	7.7%	0.0046
Anti-TNF:	0.3%	1.2%	0.194
Oral 5-ASA	83.6%	88.7%	0.1667
Smoking:			
Never Smoked:	64.5%	61.5%	0.70
Previously Smoked:	26.5%	27.2%	
Currently Smoking:	9.0%	11.2%	

A total of 38 transporter gene variants met criteria for inclusion (MAF>5% in healthy controls) and passed genotyping quality control standards. None of the transport gene variants involved in the primary analysis were associated with a response to mesalamine in univariate analysis (Table 2). A more stringent definition of mesalamine non-response was also used for subsequent analysis that required both lack of PGA improvement at Week 6 along with persistent rectal bleeding at Week 3. Despite this stricter definition of mesalamine non-response, no common transporter gene variants met the a priori threshold for significance with respect to mesalamine response. Controlling for mesalamine dose received and for patient characteristics that differed between responders and non-responders (prior immunomodulator use, prior corticosteroid use, and UC disease extent) did not identify transport gene SNPs associated with a response to mesalamine. There was not a statistically significant association between non-transport gene SNPs and mesalamine response (Table 3). The strongest non-transport gene signals were seen with rs9304334 in *ST8SIA5* (p = 1x10^-5^), rs4301242 (p = 3x10^-5^), and rs111723511 (p = 3.1x10^-5^) although they did not meet genome-wide significance thresholds.

**Table 2:** Strongest associations for common transporter gene variants with response to mesalamine. P values are represented in table were calculated by Chi square analysis with threshold for significance of 0.0013 to correct for multiple testing.

Gene	Location (Chr:BP)	Variant	MAF (Responders)	MAF (Non-Responders)	P value
*TAP2*	6:32800412	Val379Ile	0.154	0.205	0.045
*TAP2*	6:32797809	Ala565Thr	0.081	0.112	0.114
*SLCO1B1*	12:21329813	Pro155Thr	0.126	0.096	0.158
*SLC28A3*	9:86917301	Tyr113Cys	0.090	0.065	0.165
*ABCG2*	4:89052323	Gln141Lys	0.111	0.084	0.184
*SLCO1B1*	12:21331549	Val174Ala	0.193	0.225	0.239
*SLCO4A1*	20:61288038	Val78Ile	0.270	0.303	0.278
*SLCO5A1*	8:70744812	Leu33Phe	0.454	0.489	0.304
*SLC22A4*	5:131663062	Ile306Thr	0.424	0.393	0.355
*ABCC10*	6:43412865	Ile920Thr	0.228	0.202	0.358

**Table 3:** Strongest associations for genome-wide variants.

SNP	Gene	MAF (Responders)	MAF (Non-Responders)	P value
rs9304334	*ST8SIA5*	0.391	0.525	0.000010
rs4301242		0.089	0.184	0.000030
rs111723511	*C4orf19*	0.012	0.068	0.000031
rs9900486		0.296	0.418	0.000161
rs730820	*LINC01298*	0.402	0.274	0.000170
rs2714679	*LOC105375369*	0.529	0.401	0.000172
rs10890634		0.327	0.449	0.000196
rs7516189	*LOC105748977*	0.477	0.353	0.000242
rs2438466	*LINC00461*	0.154	0.251	0.000250
rs12188301		0.054	0.127	0.000270

**Fig 1:**
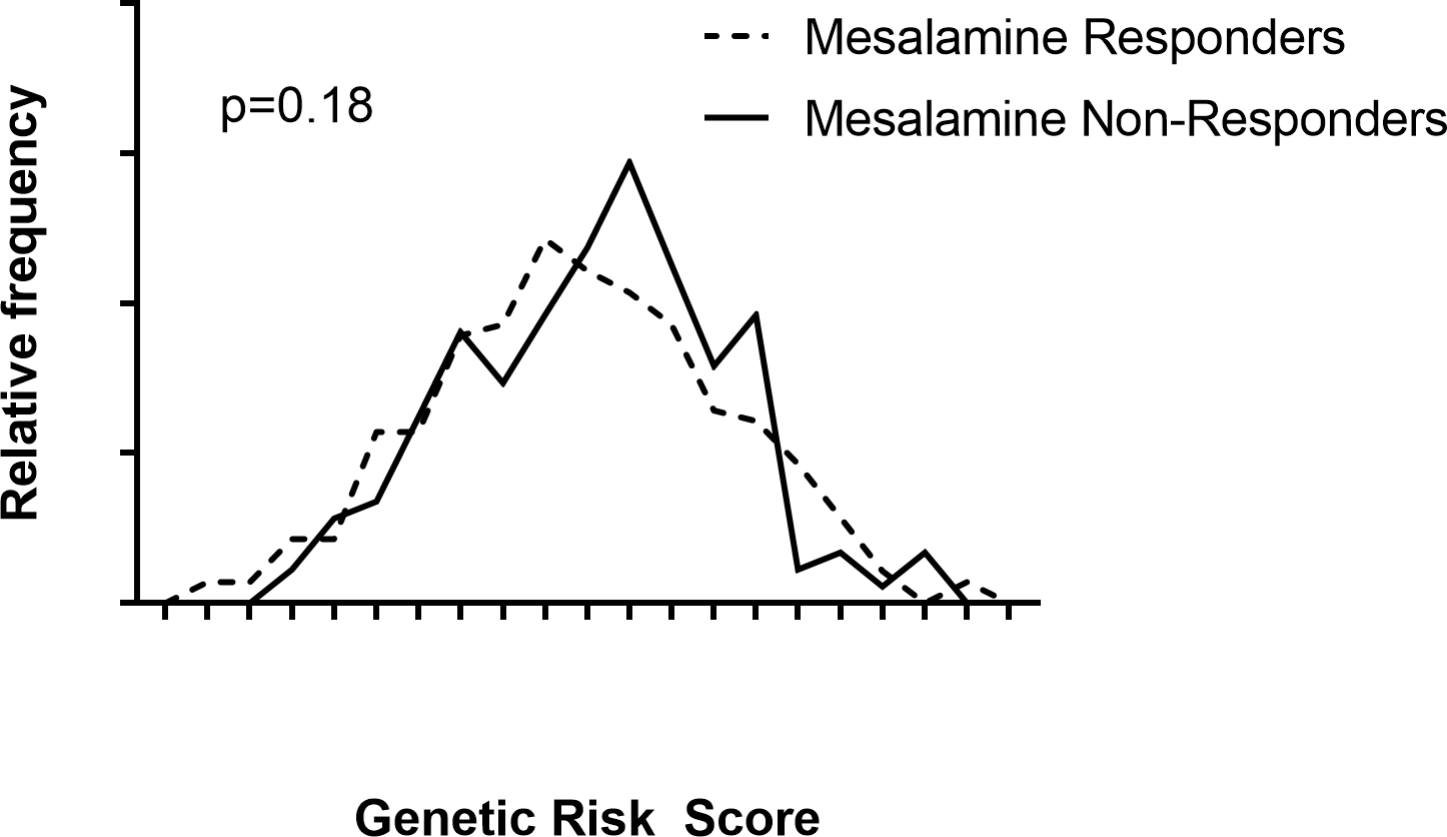
Genetic risk score distribution. **Histogram of the genetic risk scores (GRS) of mesalamine responders (dashed line) and mesalamine non-responders (solid line).** GRS was calculated by summation of the log of the odds ratio at each of 133 ulcerative colitis risk SNPs multiplied by number of risk alleles in each patient.

Genetic risk scores were calculated for all included subjects. The genetic risk score of mesalamine responders was similar to the genetic risk score of mesalamine non-responders (p = 0.18) (Fig 1). Response to mesalamine among subjects in the highest quartile of genetic risk score (58.8%) was similar to the response in the lowest quartile (64.9%, p = 0.41).

## Discussion

Mesalamine is an effective medication for the treatment of mild to moderate UC although >25% of patients fail to respond. Given that many of the potential mechanisms of action for mesalamine occur in the intracellular space and that gene variants in *SLCO1B1* affect intracellular transport of mesalamine, we sought to determine the clinical effect of variants in intestinal mucosal transport proteins.[13] In this study, these transporter variants (including those in *SLCO1B1* shown to have an in vitro effect on transport) did not have a significant effect on the efficacy of mesalamine in UC.

Mesalamine is a commonly-used treatment of UC and possesses predominantly a topical anti-inflammatory effect. The exact manner by which mesalamine works is unknown, but possible mechanisms include scavenging of reactive oxygen species, activation of PPAR-γ, inhibition of prostaglandin and leukotriene synthesis and blockade of cytokine-induced NFκB activation.[10, 11, 19] Many of the proposed mechanisms would require intracellular presence. In vitro data demonstrate that mesalamine reaches the intracellular space of epithelial cells by way of drug transporter proteins. However, the lack of clinical effect of genetic variants known to affect the transport of mesalamine into the epithelium suggests that either current dosing of mesalamine overcomes these effects on transport or that mechanisms not requiring an intracellular presence (i.e. reactive oxygen species scavenging) play a strong role in the anti-inflammatory effect of mesalamine.

This study has limitations that should be discussed. There were some differences between the mesalamine responder and non-responder groups. However, controlling for these variables did not identify any significant variants. A placebo group was not included in the design of this study because the ASCEND III trial compared two doses of mesalamine and was not placebo-controlled. Similarly, the definition of response was based on the PGA which in this case was a unique scoring system that was a combination of a clinical symptom-based index (of rectal bleeding and stool frequency) and sigmoidoscopic evaluation rather than one only based on mucosal healing. Although the inclusion of a placebo group and determination of mucosal healing may have led to the identification of a relevant genetic variant, this seems unlikely as a stricter definition of mesalamine non-response yielded similar results to the primary data analysis. Lastly, the sample size of this study would not have been afforded adequate power to detect rare variants (with MAF <5%) that might affect response to mesalamine. However, rare variants would likely contribute only a small effect on the overall response in a large population compared to the impact of these more common variants that were the focus of this study. Future studies focusing on the effect of rare variants on mesalamine efficacy would require much larger sample size than the 457 subjects in this study.

Recent observations demonstrate that the gastrointestinal microbiome plays a relevant role in drug metabolism and efficacy. Studies in a humanized mouse model have shown that digoxin is activated by *Eggerthella lenta*.[20] The concept of microbial effects of drug efficacy may have particular relevance for medications such as mesalamine which act topically in the gut and may interact with the mucosal-associated microbiome. A better understanding of the relationship between the microbiome and mesalamine could lead to prebiotic or probiotic supplements that might increase efficacy.

In this study, we demonstrated that common genetic variants in intestinal transport proteins do not affect the efficacy of mesalamine. The response to mesalamine may be due to rare genetic effects (that this study lacked the power to detect) or due to other effects such as epigenetic effects on the genome or by environmental factors such as the intestinal microbiome.

## Supporting information

S1 Table List of transporter genes in this study. (DOCX)
