# Supplementary material for: Genetic variants in cellular transport do not affect mesalamine response in ulcerative colitis
Source: PLoS One. 2018 Mar 26;13(3):e0192806. doi: 10.1371/journal.pone.0192806 (PMC5868763; doi:10.1371/journal.pone.0192806)
Supplement: S1 Table — (DOCX) [file pone.0192806.s001.docx]

Supplementary Table 1: Transporter Genes with Variants Included in Study

| ABCB1 |  |  |  |  |  |
| --- | --- | --- | --- | --- | --- |
| ABCB4 |  |  |  |  |  |
| ABCB6 ABCB7 |  |  |  |  |  |
| ABCB8 |  |  |  |  |  |
| ABCC1 |  |  |  |  |  |
| ABCC2 |  |  |  |  |  |
| ABCC3 ABCC4 |  |  |  |  |  |
| ABCC5 |  |  |  |  |  |
| ABCC6 ABCC9 |  |  |  |  |  |
| ABCC10 |  |  |  |  |  |
| ABCC11 |  |  |  |  |  |
| ABCG1 ABCG2 |  |  |  |  |  |
| SLC2A4 |  |  |  |  |  |
| SLC2A5 |  |  |  |  |  |
| SLC5A6 |  |  |  |  |  |
| SLC6A6 |  |  |  |  |  |
| SLC7A5 |  |  |  |  |  |
| SLC7A7 SLC7A8 SLC13A3 |  |  |  |  |  |
| SLC15A2 |  |  |  |  |  |
| SLC16A1 |  |  |  |  |  |
| SLC19A1 SLC22A3 |  |  |  |  |  |
| SLC22A4 |  |  |  |  |  |
| SLC22A5 |  |  |  |  |  |
| SLC22A15 SLC22A17 |  |  |  |  |  |
| SLC22A18 |  |  |  |  |  |
| SLC22A18AS |  |  |  |  |  |
| SLC27A1 |  |  |  |  |  |
| SLC28A3 |  |  |  |  |  |
| SLC29A1 |  |  |  |  |  |
| SLC29A2 SLCO1B1 SLCO1B3 |  |  |  |  |  |
| SLCO2A1 |  |  |  |  |  |
| SLCO2B1 |  |  |  |  |  |
| SLCO3A1 |  |  |  |  |  |
| SLCO4A1 |  |  |  |  |  |
| SLCO5A1 |  |  |  |  |  |
| TAP1 |  |  |  |  |  |
| TAP2 |  |  |  |  |  |
